# Addressing Cancer Disparities Through Community Engagement: Lessons and Best Practices

**DOI:** 10.7759/cureus.43445

**Published:** 2023-08-14

**Authors:** Swarali Kale, Shoyeb Hirani, Sauvik Vardhan, Aditi Mishra, Dewang B Ghode, Roshan Prasad, Mayur Wanjari

**Affiliations:** 1 Medicine, Jawaharlal Nehru Medical College, Datta Meghe Institute of Higher Education and Research, Wardha, IND; 2 Medicine, Mahatma Gandhi Mission (MGM) Medical College and Hospital, Aurangabad, IND; 3 General Surgery, Jawaharlal Nehru Medical College, Datta Meghe Institute of Higher Education and Research, Wardha, IND; 4 Medicine and Surgery, Jawaharlal Nehru Medical College, Datta Meghe Institute of Higher Education and Research, Wardha, IND; 5 Research and Development, Jawaharlal Nehru Medical College, Datta Meghe Institute of Higher Education and Research, Wardha, IND

**Keywords:** health disparities, cultural competence, community-based participatory research, lessons learned, best practices, health equity, community engagement, cancer disparities

## Abstract

Cancer disparities continue to be a significant public health challenge, disproportionately affecting certain communities in terms of incidence, mortality, and access to quality care. Addressing these disparities requires a multifaceted approach that involves not only healthcare professionals and researchers but also the active participation and collaboration of the affected communities themselves. Community engagement has emerged as a promising strategy to reduce cancer disparities and promote health equity. This review article synthesizes the existing literature and examines the role of community engagement in addressing cancer disparities. It explores various approaches and best practices utilized in community engagement initiatives to empower and involve diverse populations in the fight against cancer. The review discusses key lessons learned from successful programs and identifies challenges faced in implementing such initiatives. The article highlights the importance of cultural competence, trust-building, and meaningful collaboration between stakeholders, including community leaders, healthcare providers, researchers, and policymakers. It emphasizes the significance of tailoring interventions to specific community needs, acknowledging cultural differences, and fostering a two-way exchange of knowledge and resources. Moreover, this review investigates the impact of community engagement on cancer prevention, early detection, treatment adherence, and survivorship outcomes. It sheds light on the role of community-based participatory research and other innovative strategies in generating evidence and facilitating the translation of research findings into real-world interventions. In conclusion, this review underlines the potential of community engagement in addressing cancer disparities and promoting health equity. By involving communities as active partners in cancer control efforts, healthcare systems can design more effective and sustainable interventions. This approach not only contributes to reducing cancer disparities but also fosters a sense of ownership and empowerment within the communities affected, paving the way for a more equitable and inclusive healthcare landscape.

## Introduction and background

Cancer disparities refer to the unequal burden of cancer incidence, prevalence, mortality, and survivorship among different population groups. Various factors, including socioeconomic status, race/ethnicity, geographic location, and access to healthcare, influence these disparities. Studies have consistently shown that certain communities, such as racial and ethnic minorities, low-income individuals, and rural populations, experience higher cancer incidence rates, worse outcomes, and limited access to quality care [1-4].

Community engagement plays a crucial role in addressing cancer disparities by actively involving affected communities in designing, implementing, and evaluating initiatives and interventions. It recognizes that communities are experts in their own experiences, needs, and preferences and empowers them to actively participate in decision-making processes. Community engagement ensures that interventions are culturally appropriate, responsive, and sustainable by fostering collaborative partnerships between researchers, healthcare providers, policymakers, and community members [5].

This review article aims to explore the role of community engagement in addressing cancer disparities and highlight the lessons learned and best practices from existing initiatives. By examining successful community engagement models and strategies, this article provides insights and recommendations for researchers, healthcare providers, policymakers, and community leaders interested in developing effective approaches to reduce cancer disparities. The review will also identify challenges and limitations associated with community engagement and suggest potential future research and policy development directions in this field.

## Review

Methodology

The methodology employed for this review article involved conducting a comprehensive literature search to gather relevant information. Various electronic databases were utilized, including PubMed, Google Scholar, and academic libraries. The search strategy utilized a combination of keywords and controlled vocabulary related to cancer disparities, community engagement, and best practices. Publication dates did not limit the search to ensure a wide range of literature was considered. In selecting studies for inclusion, specific criteria were applied. Studies were included if they were relevant to the role of community engagement in addressing cancer disparities. This encompassed research articles, reviews, case studies, and reports that provided insights into community engagement initiatives, outcomes, and lessons learned. Only peer-reviewed studies published in English were considered to ensure the quality and accuracy of the information. Priority was given to studies that focused on lessons learned and best practices in community engagement, highlighting effective strategies, challenges, and recommendations. Studies were excluded if they did not meet the specified criteria or were primarily focused on theoretical frameworks or conceptual discussions without empirical evidence or practical examples. Additionally, studies that solely addressed individual-level interventions unrelated to community engagement in cancer disparities were excluded. The study selection process involved an initial screening of titles and abstracts, followed by a full-text review to assess eligibility. Disagreements were resolved through discussion and consensus among the reviewers.

Understanding cancer disparities

Definition and Scope of Cancer Disparities

Cancer disparities refer to the unequal distribution of cancer incidence, prevalence, mortality, and survivorship across different population groups. These disparities are characterized by variations in cancer outcomes based on race/ethnicity, socioeconomic status, age, gender, geographic location, and access to healthcare services. Disparities can manifest in different ways, including disparities in cancer screening and early detection, treatment utilization, access to clinical trials, and quality of care [1].

Factors Contributing to Cancer Disparities

Socioeconomic factors: Lower socioeconomic status is strongly associated with increased cancer disparities. Limited financial resources, a lack of health insurance, lower education levels, and inadequate access to healthcare facilities and services contribute to disparities in cancer prevention, detection, and treatment outcomes [6].

Racial and ethnic disparities: Racial and ethnic minorities often experience higher rates of certain cancers and poorer outcomes than non-Hispanic White populations. Factors such as systemic racism, discrimination, cultural beliefs, language barriers, and mistrust of the healthcare system contribute to these disparities [7].

Geographic disparities: Disparities in cancer incidence and outcomes are observed between urban and rural areas. Rural populations face unique challenges, including limited healthcare infrastructure, a shortage of healthcare providers, longer travel distances to access care, and reduced availability of specialized cancer services [8].

Access to healthcare: Limited access to quality healthcare services, including cancer screenings, diagnostic tests, and specialized treatments, contributes to disparities. Barriers to access may include a lack of health insurance, transportation issues, language barriers, and the inadequate availability of healthcare facilities in underserved areas [9].

Behavioral factors: Differences in health behaviors, such as tobacco use, physical inactivity, poor nutrition, and low adherence to recommended cancer screenings, contribute to disparities in cancer incidence and outcomes [10].

Impact of Cancer Disparities on Affected Communities

Cancer disparities have a significant impact on affected communities. They result in unequal disease burden, increased morbidity and mortality, decreased quality of life, and reduced survival rates for disadvantaged populations. Disparities also perpetuate social and economic inequalities, exacerbating existing health inequities. The emotional and financial toll on individuals and families affected by cancer disparities is substantial, further underscoring the urgency of addressing these disparities through targeted interventions and community engagement [11].

By understanding the factors contributing to cancer disparities and their impact on affected communities, we can develop targeted strategies and interventions that address the unique challenges different populations face. Community engagement is critical in ensuring that interventions are tailored to these communities' specific needs and contexts, ultimately leading to more equitable cancer care and outcomes [12].

Community engagement in cancer disparities

Definition and Significance of Community Engagement

Community engagement involves actively partnering with communities affected by cancer disparities in all stages of research, intervention development, implementation, and evaluation. It recognizes that community members possess valuable knowledge, experiences, and perspectives that can inform and guide efforts to address disparities. Community engagement moves beyond a passive recipient model to an active collaboration, empowering communities to be equal stakeholders in decision-making processes related to their health [13,14].

The significance of community engagement lies in its ability to ensure that interventions and strategies are culturally appropriate, relevant, and responsive to the unique needs of diverse populations. By actively involving community members, community engagement fosters trust, promotes health literacy, and enhances the acceptability and sustainability of interventions. It also facilitates the identification of community-specific barriers, strengths, and resources, leading to more effective and equitable approaches to reducing cancer disparities [15].

Benefits of Community Engagement in Addressing Cancer Disparities

Culturally appropriate interventions: Community engagement allows for developing and implementing interventions sensitive to cultural beliefs, values, and practices. By incorporating community perspectives, interventions can be tailored to overcome cultural barriers and increase acceptance and uptake among diverse populations [16].

Increased trust and engagement: Engaging communities builds trust between researchers, healthcare providers, and community members. Trust is crucial for improving health outcomes and encouraging individuals to participate in cancer prevention, screening, and treatment programs [17].

Enhanced relevance and acceptability: Community engagement ensures that interventions address the specific needs and priorities of the community. This increases the likelihood of community interventions being accepted, adopted, and sustained, leading to improved outcomes [5].

Effective dissemination of information: Community members often serve as trusted sources of information within their communities. By engaging community members, accurate and culturally relevant health information can be effectively disseminated, increasing awareness and understanding of cancer disparities and prevention measures [18].

Mobilization of community resources: Community engagement facilitates the identification and mobilization of community resources, including social support networks, community-based organizations, and local expertise. Leveraging these resources can enhance the reach and impact of interventions [19].

Role of Community Engagement in Reducing Health Inequities

Community engagement is a key strategy for reducing health inequities, including cancer disparities. By actively involving affected communities, community engagement helps to address the underlying social determinants of health, such as poverty, limited education, discrimination, and a lack of access to resources. It promotes equity by ensuring interventions are tailored to diverse populations' needs and contexts [20].

Community engagement also empowers communities, fostering a sense of ownership and agency in addressing health disparities. It encourages community members to become advocates, leaders, and partners in decision-making processes, enabling them to actively shape policies and practices that impact their health [21]. Furthermore, community engagement facilitates the implementation of comprehensive and integrated approaches that address multiple factors contributing to disparities. It promotes collaboration among various stakeholders, including community organizations, healthcare providers, policymakers, and researchers, to collectively work toward reducing health inequities.

Lessons learned from community engagement initiatives

Case Study: Community Health Workers in Cancer Prevention

This case study focuses on a community engagement initiative that utilized Community Health Workers (CHWs) to promote cancer prevention and education in underserved communities. CHWs are individuals from the community who serve as liaisons between healthcare systems and the community. They are crucial in bridging the gap by providing culturally tailored education, outreach, and support [22].

CHWs in breast cancer screening within the context of the initiative: The initiative aimed to address cancer prevention in target communities by involving the training and employment of CHWs to play crucial roles in delivering cancer prevention messages, conducting community outreach activities, and providing support to community members [23]. First, CHWs were actively engaged in education related to breast cancer screening. They played a vital role in disseminating crucial information about breast cancer, its risk factors, early detection methods, and the importance of screening. Their interactions with community members helped raise awareness and knowledge about breast cancer, empowering individuals to make informed decisions about their health. Second, CHWs provided direct assistance in breast cancer screening. As trained and trusted community members, they offered practical support in facilitating the screening process. This involved guiding individuals through getting screened, helping them schedule appointments, and addressing any concerns or fears related to the screening procedure. Their presence eased the process for many community members, increasing participation in breast cancer screening. Lastly, CHWs performed navigational services as a bridge between community members and healthcare facilities. They understood the cultural context and language of the community, enabling them to ensure that educational materials and messages about breast cancer were culturally appropriate and relevant. Additionally, they helped community members navigate the healthcare system, facilitating access to screening facilities, diagnostic tests, and further medical care when needed. Overall, the involvement of CHWs in the initiative played a pivotal role in enhancing breast cancer screening rates in the target communities. By focusing on education, direct assistance, and navigational services, they significantly contributed to promoting early detection and reducing barriers to breast cancer screening, ultimately improving the overall health outcomes in the communities they served [23].

The significance of this initiative's impact was rigorously assessed through a comprehensive evaluation process. To determine the effectiveness of the intervention, statistical analyses were conducted, employing both parametric and non-parametric tests, such as t-tests and chi-square tests, respectively. These tests were utilized to compare pre-and post-intervention data, assessing changes in cancer awareness levels, understanding of risk factors, and uptake of preventive measures among community members. Awareness levels were measured through pre- and post-intervention surveys, where participants were asked about their knowledge of cancer risk factors and preventive measures. A significant improvement in awareness was observed, with a considerable increase in correct responses in the post-intervention survey compared to the baseline. Quantitative data on the number of individuals undergoing cancer screening before and after the intervention was collected to evaluate the impact on screening rates. Additionally, qualitative assessments were conducted through focus group discussions and interviews with community members to understand their perceptions of the initiative and the factors influencing their decision to seek cancer screening services. The involvement of CHWs was particularly instrumental in creating a supportive and culturally sensitive environment, encouraging community members to feel more comfortable accessing screening services, which, in turn, contributed to the observed increase in screening rates. Moreover, preventive measures were assessed through community-level data on adopting healthier lifestyles and behaviors, such as tobacco cessation, dietary improvements, and increased physical activity. These measures were incorporated into the initiative with the support of CHWs, who played a pivotal role in promoting behavioral changes and providing continuous guidance to community members. As a result of this comprehensive initiative, notable reductions in cancer outcome disparities were observed within underserved communities. The combined effect of increased awareness, improved screening rates, and the adoption of preventive measures contributed to narrowing the gap in cancer outcomes between underserved communities and other populations [24].

Case Study: Culturally Tailored Cancer Education Programs

This case study examines a community engagement initiative that developed culturally tailored cancer education programs for specific ethnic or cultural communities. Recognizing the importance of cultural beliefs, values, and language preferences in effective communication, this initiative aimed to adapt educational materials and messages to align with the specific cultural context of the target communities [25].

The study highlighted a concerning lack of general awareness about cancer among the Indian population, particularly concerning its curability, preventability, and available screening methods. Our research indicated that education and place of residence (rural or urban) play pivotal roles in shaping cancer awareness. Notably, awareness of risk factors was predominantly limited to tobacco and alcohol consumption. On a positive note, we observed a favorable attitude toward screening modalities among the Indian population. However, despite this positive attitude, actual screening practices were found to be poor. The study identified the potential benefit of creating community-level awareness to enhance screening practices. The initiative focused on close collaboration with community members, cultural leaders, and healthcare providers to address this. Educational materials were thoughtfully designed to be culturally sensitive and linguistically accessible, incorporating familiar symbols, traditions, and real-life examples that resonated with the community's beliefs and experiences [26]. This culturally tailored approach aimed to bridge the knowledge gap and promote positive health-seeking behavior in cancer screening. By adopting such community-specific strategies and interventions, there is an opportunity to improve screening practices and subsequently reduce the burden of cancer in the Indian population. Understanding the cultural context and actively engaging with the community can prove instrumental in fostering greater awareness and encouraging timely screenings, leading to more effective cancer prevention and early detection efforts.

The positive outcomes of this initiative were notable. The culturally tailored cancer education programs increased awareness of cancer prevention, screening, and early detection within the target communities. Community members felt more empowered to take proactive steps toward cancer prevention and engage in regular screenings. This initiative also fostered a sense of community empowerment as individuals felt that their cultural values and perspectives were valued and included in the educational materials [27].

By highlighting these case studies, it becomes evident that community engagement initiatives, such as employing CHWs and developing culturally tailored programs, have effectively addressed cancer disparities. These examples underscore the importance of adapting interventions to specific community contexts, leveraging cultural strengths, and actively involving community members in the design and implementation of initiatives [28].

Evaluation of outcomes and impact of community engagement

Measurement of Intervention Outcomes

Evaluating community engagement initiatives involves employing various evaluation methodologies to assess the outcomes and impact of these interventions. Quantitative and qualitative measures are utilized to comprehensively understand community engagement's effects on addressing cancer disparities [29].

Quantitative measures may include assessing changes in cancer knowledge, behavior, screening rates, and access to care. For example, surveys or questionnaires can measure changes in cancer knowledge and screening behaviors before and after community engagement interventions. Data on screening rates and access to care can be collected from healthcare facilities or registries to evaluate the impact of community engagement on these outcomes [30].

Qualitative measures capture the experiences, perspectives, and stories of community members and stakeholders involved in community engagement initiatives. Interviews, focus groups, or qualitative surveys can be conducted to explore the qualitative impact of community engagement on individual and community-level outcomes. These measures provide insights into participants' lived experiences and allow for a deeper understanding of the effects of community engagement [31]. It is essential to incorporate community-defined indicators and outcome measures in the evaluation process. Community members should be actively involved in identifying meaningful indicators that reflect their priorities and values. This ensures that the evaluation captures the most relevant and important outcomes to the community, providing a comprehensive assessment of the impact of community engagement initiatives.

Impact on Disparities Reduction

Community engagement initiatives have demonstrated their potential to reduce cancer disparities significantly. These initiatives are instrumental in addressing the underlying factors contributing to disparities and are pivotal in promoting equitable access to cancer prevention, screening, and treatment services [32]. Participants in such initiatives have strongly preferred a community outreach approach to cancer screening, particularly favoring mobile community-based screening and community information sessions. Adopting an ethno-specific lens when tailoring these initiatives to the targeted communities is important. The feedback from participants highlighted the efficacy of models of primary care that extend support to the entire local community and offer some of their services directly within that community. Such an approach can potentially make a meaningful impact on cancer screening, especially for socially marginalized groups. By embedding screening services within the community, individuals facing barriers to healthcare access can benefit from more convenient and approachable avenues for early cancer detection. Mobile community-based screening units have emerged as a practical and efficient means of reaching underserved populations. These units can overcome geographical barriers, bringing cancer screening services directly to remote or isolated communities. Moreover, community information sessions have proven powerful tools for increasing awareness and knowledge about cancer prevention and screening. By actively engaging community members in these sessions, the initiatives can dispel misconceptions, enhance health literacy, and foster a sense of empowerment among participants [32]. 

One of the significant impacts of community engagement is the improvement in screening rates among marginalized populations. Community engagement interventions focusing on education, outreach, and navigation services have successfully increased awareness and utilization of cancer screenings. By tailoring interventions to the community's specific needs and cultural contexts, community engagement can effectively address barriers and increase screening rates among underserved populations [33].

Additionally, community engagement initiatives have played a crucial role in increasing access to care. By actively involving community members in decision-making processes and partnering with local healthcare providers, these initiatives have facilitated developing and implementing programs that address healthcare access barriers. By addressing these barriers, community engagement interventions have improved access to timely and appropriate cancer care, reducing disparities in treatment and healthcare outcomes [34].

Identification of key lessons learned from these initiatives

Building Trust and Partnerships

Establishing trusting relationships and partnerships with community members, organizations, and stakeholders is crucial to community engagement initiatives. Lessons learned emphasize the importance of fostering mutual respect, transparency, and effective communication to ensure meaningful engagement. Building trust involves actively listening to community concerns, addressing power dynamics, and demonstrating a commitment to collaboration. By prioritizing relationship-building and trust, community engagement initiatives can lay a strong foundation for successful interventions [35].

Culturally Appropriate Approaches

Tailoring interventions to the community's cultural, linguistic, and social contexts is essential to community engagement. Lessons learned highlight the significance of adapting educational materials, messages, and interventions to align with community values, practices, and beliefs. This involves understanding the community's cultural nuances, language preferences, and traditional practices. By incorporating cultural competency, interventions become more relatable, acceptable, and effective in addressing cancer disparities [36].

Capacity Building and Empowerment

A key lesson learned in community engagement initiatives is empowering community members through education, training, and leadership development. Supporting community-driven initiatives, mobilizing resources, and fostering sustainability beyond the duration of specific projects are important aspects. By providing opportunities for community members to build their skills, knowledge, and leadership capacity, they become active participants in decision-making processes and drivers of change within their communities. Empowerment fosters ownership, sustainability, and long-term impact in addressing cancer disparities [37].

Advocacy and Policy Change

Lessons learned highlight the value of leveraging community engagement to advocate for policy changes that address systemic barriers contributing to cancer disparities. Community engagement allows community members to share their experiences and perspectives, influencing policy decisions. Examples of successful advocacy efforts resulting in policy changes that have positively impacted cancer outcomes in marginalized communities serve as inspiration. By engaging in advocacy and policy change, community engagement initiatives can create systemic changes that address the root causes of cancer disparities [38].

Best practices for effective community engagement

Developing Strong Partnerships With Community Organizations

Developing strong partnerships with community organizations is a cornerstone of effective community engagement. This involves fostering collaborative partnerships with community-based organizations, community leaders, and stakeholders. It is essential to involve them in decision-making to ensure their active participation in identifying needs, setting goals, and implementing interventions [39].

Collaboration and co-creation are key principles in building partnerships. By working collaboratively with community organizations, researchers, healthcare providers, and policymakers can tap into these organizations' knowledge, expertise, and resources. This collaborative approach allows for a comprehensive understanding of the community's unique challenges and strengths, leading to the development of targeted interventions that address their specific needs [40].

Shared leadership is another vital element in developing strong partnerships. Recognizing and valuing community partners' expertise, knowledge, and experiences is important. Actively involving them in all aspects of the engagement process, from planning to implementation and evaluation, ensures that decision-making is inclusive and representative. By embracing shared leadership, power dynamics within partnerships become more equitable, allowing for a more balanced distribution of responsibilities and decision-making authority [41].

Cultivating long-term relationships with community organizations is crucial for building trust and continuity. It requires a commitment to sustained collaboration beyond the duration of specific projects or research studies. Long-term engagement allows for deeper connections, allowing time to establish rapport, understand community dynamics, and develop mutual trust. Building long-lasting relationships with community organizations creates a foundation for ongoing collaboration, enabling a more meaningful and sustained impact on addressing cancer disparities [42].

Cultural Competence and Sensitivity in Community Engagement

To effectively engage with communities, it is crucial to invest in developing cultural competence among researchers, healthcare providers, and professionals involved in community engagement. This involves understanding the community's cultural values, practices, and beliefs to ensure that interventions are respectful, relevant, and appropriate. Cultural understanding helps to avoid assumptions and stereotypes, allowing for a more meaningful and impactful engagement process [43].

In addition to cultural understanding, linguistic accessibility is vital for effective community engagement. It is essential to provide materials, resources, and communication in languages spoken by the community. Language barriers can hinder effective communication and understanding. Professional interpreters or bilingual staff can bridge these language barriers, ensuring community members accurately convey and understand information. Linguistic accessibility fosters inclusivity and promotes equal participation in community engagement [44].

Furthermore, tailoring interventions to align with the cultural context and preferences of the community is essential. By adapting interventions to the community's cultural context, interventions become more relatable and acceptable. This can involve incorporating culturally specific approaches and practices that resonate with community members. Tailoring interventions acknowledges and respects the diverse cultural backgrounds of the community, increasing the likelihood of community acceptance and engagement [45]. By investing in cultural understanding, linguistic accessibility, and tailoring interventions, community engagement efforts can effectively address cancer disparities. These practices foster a sense of respect, relevance, and cultural sensitivity, enhancing the overall effectiveness of interventions. They ensure community members feel heard, valued, and included in decision-making, leading to more meaningful and sustainable community engagement initiatives.

Strategies for Effective Communication and Collaboration

Clear and transparent communication is essential for effective community engagement. Using plain language and clear communication techniques helps ensure the community easily understands information. It is important to convey project goals, methodologies, and expected outcomes in a manner that is accessible to all community members. Transparency should be maintained throughout the engagement process, fostering trust and ensuring community members are well-informed about the initiatives' purpose, progress, and potential impact [46].

Active listening is a key component of successful community engagement. It involves genuinely understanding the community's needs, concerns, and aspirations. Community members' voices are valued and respected by creating spaces for community members to share their experiences, perspectives, and suggestions. Active listening enables a deeper understanding of the community's unique challenges, strengths, and preferences, which can inform the development of interventions that are more responsive and relevant [47].

Flexibility and adaptability are crucial when engaging with communities. Being open to adapting strategies and interventions based on community feedback and evolving needs is essential for success. Communities are dynamic, and their needs may change over time. By remaining flexible, engagement efforts can be adjusted to address emerging issues, incorporate community feedback, and ensure that interventions are tailored to specific contexts. This approach increases the effectiveness and acceptance of interventions by demonstrating a commitment to responding to unique circumstances and evolving community needs [48].

Building Trust and Meaningful Relationships With Community Members

Respect and empathy are essential when approaching community engagement initiatives. It is crucial to approach communities with genuine respect for their knowledge, experiences, and cultural diversity. Recognizing the community's strengths, resilience, and expertise fosters a collaborative and empowering environment. By valuing and amplifying the voices of community members, their contributions become integral to decision-making processes and the development of interventions [49].

Transparent and ethical practices are paramount in community engagement. Researchers and practitioners must maintain ethical standards throughout the research and engagement processes. This includes ensuring transparency in data collection, use, and dissemination. Obtaining informed consent from community members participating in research is essential to protecting their rights and privacy. Respecting ethical guidelines builds trust, credibility, and mutual understanding between researchers, practitioners, and the community [50].

Consistency and reliability are key factors in building trust and maintaining meaningful engagement. Demonstrating consistency in engagement efforts, including regular communication and updates, helps foster a sense of reliability and accountability. Upholding commitments and honoring agreements made with the community is essential to building trust and sustaining the partnership. By providing ongoing updates, community members feel valued, informed, and engaged, reinforcing trust and commitment between all parties involved [51].

Empowering Communities through Education and Resources

Empowering communities through education and resources is crucial to community engagement in addressing cancer disparities. This involves providing educational resources and training opportunities to enhance health literacy, empower community members to make informed decisions and build their capacity for active participation [52].

Education and skill building are vital to empowering community members to take control of their health. By providing educational resources tailored to the community's specific needs and cultural context, individuals can learn about cancer prevention, early detection, treatment options, and survivorship. This empowers them to make informed decisions and take proactive steps toward better health outcomes [5]. In addition to education, ensuring access to resources is essential for reducing cancer disparities. Identifying and addressing barriers to accessing resources and services is crucial. This includes advocating for equitable access to quality healthcare, cancer screenings, treatment options, and supportive care services. By addressing structural and systemic barriers, such as financial constraints, transportation issues, and language barriers, communities can overcome obstacles to receiving the necessary care.

Sustainable empowerment is another key aspect of community engagement. It involves facilitating community-led initiatives and supporting the development of community-driven solutions. Selflessness and sustainability can be fostered by empowering community leaders and organizations. This may involve providing resources, mentorship, and guidance to support the development and implementation of community-led interventions. By empowering communities to take ownership of their health, long-term impacts can be achieved beyond the duration of specific projects or interventions. Through education, access to resources, and sustainable empowerment, community engagement initiatives can empower communities to actively participate in addressing cancer disparities. By equipping individuals with knowledge, resources, and the skills needed to advocate for their health, long-lasting changes can be achieved, ultimately leading to improved cancer outcomes and reduced disparities.

Challenges and limitations of community engagement

Barriers to Community Engagement in Addressing Cancer Disparities

Limited resources: Insufficient funding, staffing, and infrastructure pose significant barriers to community engagement initiatives. A lack of resources can impede the capacity to sustain long-term partnerships and adequately support community-driven initiatives. Adequate funding and resources are essential to foster meaningful collaboration and ensure the sustainability of community engagement efforts [46].

Mistrust and historical trauma: Historical experiences of mistreatment, discrimination, and research abuses have contributed to a deep-rooted mistrust of researchers and healthcare systems among certain communities. Building trust and overcoming this historical trauma are crucial for successful community engagement. It requires transparency, cultural sensitivity, and a commitment to addressing past injustices [53].

Time and commitment: Effective community engagement requires significant time, effort, and sustained commitment. Engaging communities in decision-making processes, maintaining open lines of communication, and involving them throughout the entire engagement process can be challenging for researchers and healthcare professionals, who may face competing priorities and time constraints. However, prioritizing and allocating sufficient time and resources to community engagement efforts is critical for their success [54].

Language and cultural barriers: Language and cultural differences can challenge effective communication and understanding between researchers, healthcare providers, and community members. It is crucial to overcome these barriers through interpreters, culturally adapted materials, and culturally competent approaches. By addressing language and cultural differences, meaningful engagement can occur, ensuring community members' voices are heard and understood [55].

Diverse community needs: Communities affected by cancer disparities have unique needs, preferences, and priorities. Developing interventions that effectively address a community's diverse needs can be challenging. It requires a deep understanding of the community's cultural, social, and economic contexts and implementing tailored approaches responsive to these diverse needs. Engaging community members in designing and implementing interventions can help ensure their relevance and effectiveness [56].

Overcoming Challenges and Limitations

Community-driven approaches: Emphasizing community leadership and involvement in decision-making is crucial for effective community engagement. Engaging communities as equal partners allows them to have a voice and actively shape the direction and implementation of interventions. Community members are experts in their own experiences and have valuable insights into the challenges and solutions that are most relevant to their communities. Empowering communities to take ownership of interventions fosters a sense of ownership and commitment, leading to increased acceptance, sustainability, and, ultimately, better outcomes [57].

Culturally tailored strategies: Recognizing and respecting the cultural context, language preferences, and community-specific needs is essential to community engagement. Interventions should be tailored to align with the cultural nuances and values of the community. Employing cultural brokers or community health workers who deeply understand the community's cultural norms and customs can ensure that interventions are culturally sensitive and appropriate. By incorporating culturally tailored strategies, interventions become more accessible, relatable, and effective, leading to increased engagement and better outcomes [58].

Building trust and relationships: Building trust is foundational to successful community engagement. Transparency, honest communication, and consistent engagement are keys to establishing and maintaining trust with community members. Investing time and effort in building relationships allows for open dialogue, shared understanding, and mutual respect. Long-term partnerships demonstrate commitment and reliability, reinforcing trust over time. Trust is crucial for community members to feel comfortable sharing their perspectives, concerns, and ideas, leading to more meaningful collaborations and successful interventions [58].

Capacity building: Investing in the capacity building of community members is essential for sustainable community engagement. By providing training, education, and skill development opportunities, community members gain the knowledge and tools to actively participate in initiatives. Empowering community leaders to take ownership of interventions promotes sustainability beyond the duration of specific projects. Building community capacity ensures communities have the skills, resources, and confidence to continue addressing cancer disparities in the long run [5].

Flexibility and adaptability: Flexibility and adaptability are vital in community engagement, as needs and circumstances may change over time. Remaining flexible allows for adjustments in strategies and approaches based on community feedback and emerging challenges. Continuously assessing and responding to the community's evolving needs ensures that interventions remain relevant and effective. Being open to feedback, learning from mistakes, and adapting accordingly demonstrate a commitment to meeting the community's evolving needs and improving outcomes [59].

Addressing Ethical Considerations in Community-Engaged Research

Ethical considerations are of paramount importance in community-engaged research and engagement activities. Several key aspects should be emphasized to ensure ethical practices throughout the process. First, informed consent is essential. Community members participating in research and engagement activities should provide informed consent, understanding the purpose, risks, benefits, and privacy considerations involved. It is crucial to communicate this information in a culturally appropriate and accessible manner, enabling individuals to make informed decisions about their participation [60].

Confidentiality and privacy protection are vital to trust and respect within community engagement initiatives. Safeguarding the confidentiality of community members' information is crucial, and protocols should be established to handle data securely and ethically. This includes implementing appropriate data storage and management practices to ensure that personally identifiable information is kept confidential and that privacy is respected throughout the research process. Addressing power imbalances is essential for ethical community engagement. Researchers should recognize and actively work to mitigate power differentials between themselves and community members. A collaborative approach that values community expertise, decision-making autonomy, and shared leadership is important. This ensures the community's voice is heard, respected, and incorporated into the research and engagement process [61].

Community benefits should be a central consideration in community engagement initiatives. Beyond research participation, these initiatives should strive to provide tangible benefits to the community. This may include improving access to care, increasing health literacy, or offering capacity-building opportunities. By focusing on community benefits, ethical engagement practices promote the well-being and empowerment of the community beyond the immediate research objectives [5]. Lastly, continuous ethical reflection is crucial throughout the research and engagement processes. Engaging in ongoing dialogue with community members allows for regular assessment of ethical implications. Ethical reflection involves critically examining the potential impact of research and engagement activities on the community, continually evaluating the ethical considerations at play, and making necessary adjustments to protect community interests.

Future directions and recommendations

Areas for Further Research and Investigation

Long-term impact evaluation is essential to assess the sustained effects of community engagement interventions on cancer disparities. Conducting evaluations over an extended period allows for a comprehensive understanding of the long-term outcomes of cancer outcomes, healthcare utilization, and health equity. This evaluation can provide valuable insights into the effectiveness and durability of community engagement approaches to reducing disparities. Investigating the intersectionality of multiple social determinants of health and disparities is crucial. Understanding how factors such as race, socioeconomic status, gender, and geographic location intersect to influence cancer outcomes is essential for developing targeted interventions. By exploring these intersections, researchers can identify specific populations' unique challenges and needs and design interventions that address the complex interplay of multiple disparities.

The potential of innovative technologies, such as telehealth, mobile applications, and digital platforms, to enhance community engagement in cancer care, education, and support should be explored. Assessing their effectiveness and acceptability among diverse populations is important to ensure equitable access to technological advancements. Innovative technologies have the potential to enhance communication, education, and support for underserved communities, improving their engagement in cancer prevention, treatment, and survivorship. Fostering participatory and community-led research approaches empowers communities to actively participate in defining research questions, methodologies, and outcomes. Collaborating with community members throughout the research process enhances relevance, inclusivity, and cultural appropriateness. Community-driven research ensures that the priorities and perspectives of the community are incorporated, resulting in more meaningful and impactful research outcomes.

Policy Implications and Advocacy for Community Engagement

First, policy integration is essential. Advocating for integrating community engagement approaches into cancer-related policies, guidelines, and funding mechanisms helps ensure that community participation is prioritized in decision-making processes. By highlighting the importance of community involvement, policies can address cancer disparities more effectively and promote equitable outcomes. Securing funding support for community engagement initiatives is vital for their success and sustainability. Advocacy efforts should focus on increasing funding for these initiatives and ensuring sustained resources to support collaboration, capacity building, and community-led interventions. Promoting funding opportunities that prioritize projects with community engagement components can help establish and strengthen community-driven interventions.

A health equity focus is necessary for policy initiatives. Advocacy efforts should call for policies prioritizing health equity and addressing the root causes of cancer disparities. These policies should promote equal access to quality healthcare, cancer prevention, and screening programs. By addressing systemic barriers and promoting an equitable distribution of resources, policies can help reduce disparities and improve cancer outcomes for marginalized populations. Encouraging the establishment of collaborative networks is another important advocacy effort. These networks should include researchers, healthcare providers, policymakers, and community organizations. They facilitate knowledge exchange, resource sharing, and dissemination of best practices. Collaborative networks promote synergy and collective action, allowing stakeholders to learn from one another, share successful strategies, and work together toward reducing cancer disparities.

Recommendations for Integrating Community Engagement in Cancer Care and Research

First, institutional support is crucial. Institutions should prioritize community engagement by allocating resources and establishing dedicated positions or departments focused on it. This ensures that individuals and teams are responsible for coordinating and implementing community engagement initiatives. Furthermore, providing training and support for researchers and healthcare providers in community engagement principles and practices is essential to equip them with the necessary skills and knowledge. Education and training play a vital role in promoting effective community engagement. Incorporating community engagement principles and practices into educating and training healthcare professionals, researchers, and public health practitioners is important. This includes fostering an understanding of the significance of cultural competence, ethics, and community partnerships. By integrating these concepts into formal education programs, future healthcare professionals and researchers will be better prepared to engage with communities in a culturally sensitive and ethical manner.

Collaborative platforms are essential for facilitating effective community engagement. Developing platforms that enable collaboration, knowledge sharing, and best practice dissemination among researchers, healthcare providers, and community organizations is crucial. These platforms can be networks, forums, or online communities promoting ongoing dialogue, exchanging ideas, and sharing experiences and resources. By fostering collaboration and communication, these platforms enhance the collective understanding and implementation of community engagement initiatives. Continuous evaluation and learning are vital to the success of community engagement efforts. It is important to continuously evaluate community engagement initiatives' impact and outcomes, learning from successes and challenges. This evaluation process allows for the identification of effective strategies and areas for improvement. Findings and lessons learned should be shared widely within the research and healthcare communities to inform future efforts and contribute to the evidence base of effective community engagement approaches.

## Conclusions

In conclusion, community engagement is a powerful strategy for addressing cancer disparities. By actively involving communities in all stages of research, intervention development, and evaluation, we can create meaningful and sustainable solutions. This review highlighted key points, including the importance of strong partnerships with community organizations, cultural competence, effective communication, trust-building, and community empowerment through education and resources. Ongoing community engagement efforts are crucial in the fight against cancer disparities. Through collaboration and shared decision-making, we can develop interventions tailored to diverse populations' unique needs and contexts. By fostering trust, we can bridge the gap between healthcare systems and communities, leading to improved acceptance and uptake of interventions. Empowering communities through education and resources ensures long-term sustainability and equips individuals with the tools to make informed decisions about their health. A call to action is needed to prioritize community engagement in cancer care and research. Researchers, healthcare providers, policymakers, and community leaders must recognize the transformative potential of community engagement and actively support its implementation. This includes advocating for policies promoting equitable access to care, funding initiatives prioritizing community engagement, and integrating community perspectives into decision-making processes. By working together, we can address cancer disparities, promote health equity, and create a future where every community has equal opportunities for optimal cancer prevention, treatment, and survivorship.
